# Burkitt’s lymphoma of the jaws in the Amazon region of Brazil

**DOI:** 10.4317/medoral.18936

**Published:** 2013-08-29

**Authors:** Hélder A. Rebelo-Pontes, Michelle C. de Abreu, Douglas M. Guimarães, Felipe P. Fonseca, Bruno AB. de Andrade, Oslei P. de Almeida, Décio S P. Júnior, Flávia S. Corrêa-Pontes

**Affiliations:** 1 DDS, PhD. DDS. DDS. DDS, MSc . DDS, PhD. João de Barros Barreto University Hospital, Federal University of Pará, Pará, Brazil; 2 DDS, MSc. DDS, PhD. Piracicaba Dental School, State University of Campinas, São Paulo, Brazil; 3 DDS, PhD. Department of Stomatology (Oral Pathology), Dental School, University of São Paulo, São Paulo, Brazil

## Abstract

Objectives: To describe the clinicopathologic and immunohistochemical features of Burkitt’s lymphoma of the jaws in 7 patients of Northern Brazil. Study Design: Clinical data concerning gender, age, affected site, clinical presentation, symptomatology and follow-up were collected from the clinical files. Histopathology was complemented with a broad immunohistochemical panel and in situ hybridization for Epstein-Barr virus (EBV). Results: Most of the patients were infants and 5 out of 7 were males. The mandible was affected in 5 cases and all patients also presented abdominal involvement. All cases were positive for CD45, CD20, CD79a, CD10, Bcl-6 and EBV. Ki-67 proliferative index was approximately 100%. Six patients were treated with R-CHOP (Rituximab + Cyclophosphamide, Doxorubicin, Vincristine and Prednisolone) chemotherapy, and 2 of these died of the disease. One young adult patient refused treatment and died 3 months after initial diagnosis. Conclusions: Burkitt’s lymphoma of the jaws diagnosed in the Amazon region of Brazil present similar clinicopathologic features to those described in endemic areas of Africa, including EBV positivity.

** Key words:**Burkitt’s lymphoma, EBV, Brazil, Amazon region.

## Introduction

Burkitt’s lymphoma (BL) is a B-cell lineage neoplasia first reported by the British physician and missioner Dr. Albert Cook in Uganda in the beginning of the twentieth century, followed by a more complete description by the Irish surgeon Dr. Dennis Burkitt. It was originally described as an aggressive tumor that frequently affected the gnathic bones of children from equatorial Africa, whose lymphoid origin was proposed in 1960 by the pathologist George O´Conor (1-6). BL presents important biological characteristics, since it was one of the first human tumors to be consistently associated with a viral infection, to present a chromosomal translocation that activates an oncogene (c-MYC) and to be correlated with human immunodeficiency virus (HIV) infection (5).

Currently, the World Health Organization classiﬁes BL in three main variants: endemic, sporadic and immunodeﬁcien-cy-associated types. The endemic form frequently involves the jaw bones and the abdomen of equatorial African children, whereas the sporadic form usually presents as an abdominal mass in adult patients from North America and Europe. The immunodeﬁciency-associated variant has a similar clinical presentation as that of sporadic subtype, with rare orofacial involvement (7).

The Northern region of Brazil, corresponding to the Amazon forest, with high temperatures and frequent rainfalls during the whole year, together with a poor social condition and lack of basic health and hygienic public facilities, presents an environment similar to those observed in the African BL belt area. BL characteristics have been described in different areas of Brazil (8-13), but a more detailed description of cases affecting patients from the Amazon region remains to be fully investigated, particularly those affecting the jaws. Therefore, the aim of this study is to describe the clinicopathological, immunohistochemical and Epstein-Barr virus (EBV) status of BL affecting the gnathic bones of 7 patients from Northern Brazil and compare the results with other reports from Brazil and endemic areas of Africa.

## Material and Methods

In a 5-year period from July 2007 to August 2012, cases diagnosed as BL involving the jaws were retrieved from the archives of the Service of Oral Pathology of the João de Barros Barreto University Hospital. The original H&E stained slides of each case were reviewed by two oral pathologists and 3µm sections were submitted to immunohistochemical reactions using a large panel of markers. In brief, sections were de-waxed with xylene and hydrated in an ethanol series. After antigen retrieval, endogenous peroxidase activity was blocked using 10% hydrogen peroxide. After washing in phosphate buffered saline (PBS) buffer (pH 7.4), slides were incubated overnight with primary antibodies ( Table 1) and subsequently exposed to DAKO labeled streptavidin biotin (LSABTM) Kit (DakoCytomation, USA) and diaminobenzidin tetrahydrochloride (DAB, Sigma, St. Louis, MO, USA), and subsequently counterstained with Carazzi hematoxylin.

**Table 1:**
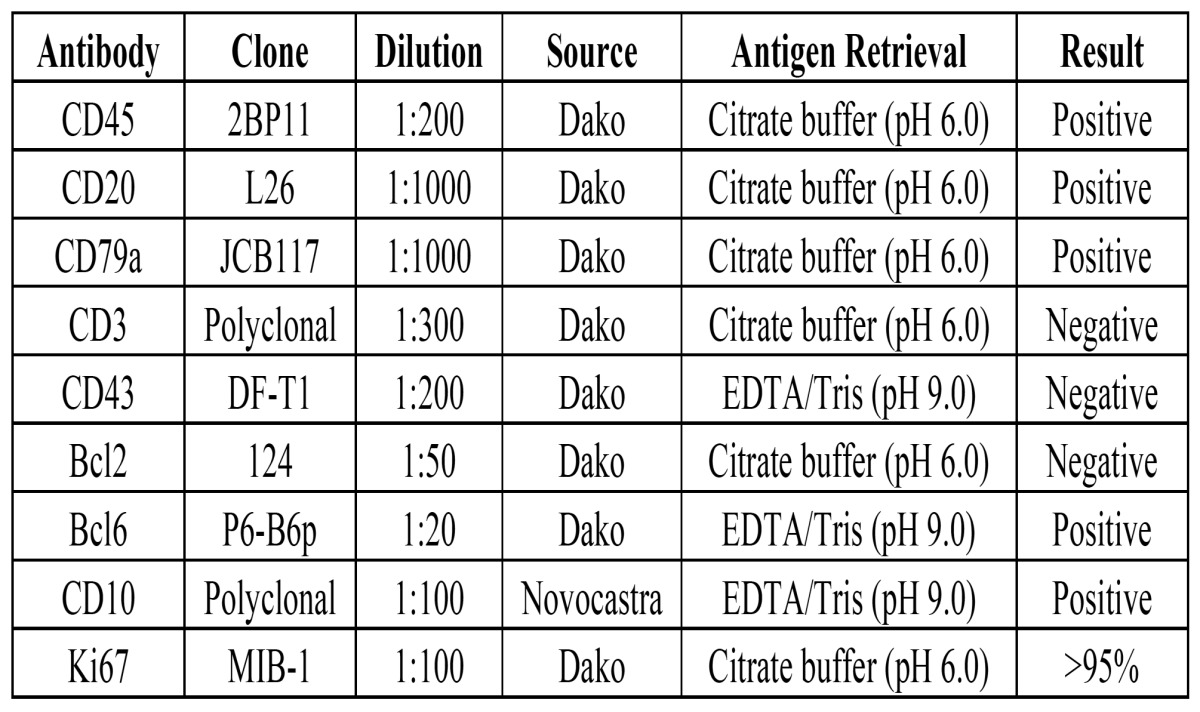
Immunohistochemical features found in 7 cases of Burkitt’s lymphoma affecting the jaws of patients from the Amazon region of Brazil.

In situ hybridization (ISH) staining for Epstein–Barr virus-encoded small nuclear RNA (EBER) was performed using peptide nucleotide acid (PNA) probes conjugated with 5-carboxiﬂuorescein complementary to EBER1 and EBER2 loci and substrate 5-Bromo-4-chloro-3-indolyl-phosphate (BCIP)/ nitro blue tetrazolium (NBT) (EBER, PNA probes; DakoCytomation, USA).

Clinical data were collected from the medical records of the patients and comprised informations about gender, age, affected site, HIV infection status, symptoms, treatment modality and follow-up. All patients were investigated for involvement of other sites, including abdomen, by ultrasound and computed tomography.

## Results

**Figure 1:**
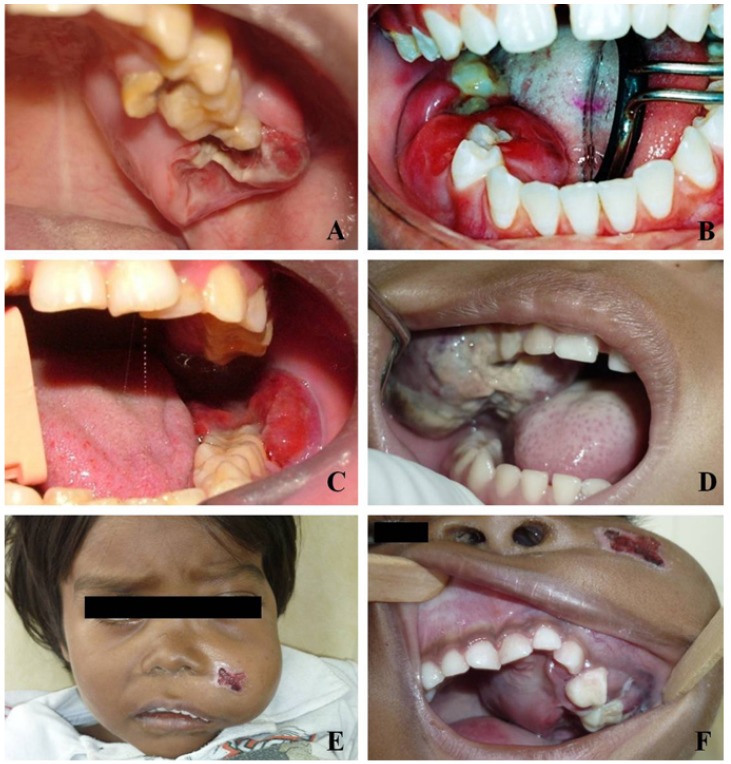
Clinical presentation of BL involving the jaws of patients from the Amazon region of Brazil. A) BL involving the maxilla of a 19 years old male patient. B and C) BL affecting the posterior areas of the mandible of an 8 and 9 years old male patients, respectively. D) BL involving the maxilla of a 5 years old male patient with extensive areas of necrosis. E and F) BL involving the maxilla and causing facial asymmetry and cutaneous ulceration in a 3 years old male patient.

The main clinical features of the seven cases of BL from the Northern region of Brazil, affecting the gnathic bones are shown in  Table 2. Five of the patients were males, with age ranging from 3 to 54 years old, with a mean of 17.1 years. Five patients presented only one oral lesion, three affecting the posterior mandible and two the posterior region of the maxilla, whereas the two other cases involved both the mandible and maxilla. Facial asymmetry was observed in three patients, one showing bilateral swelling and one cutaneous ulceration (Fig. 1). All patients presented tooth mobility and alveolar bone destruction with root resorption and lamina dura loss in the affected site. Abdominal involvement was found in all patients, and four reported pain. None of the patients was HIV positive.

**Table 2:**
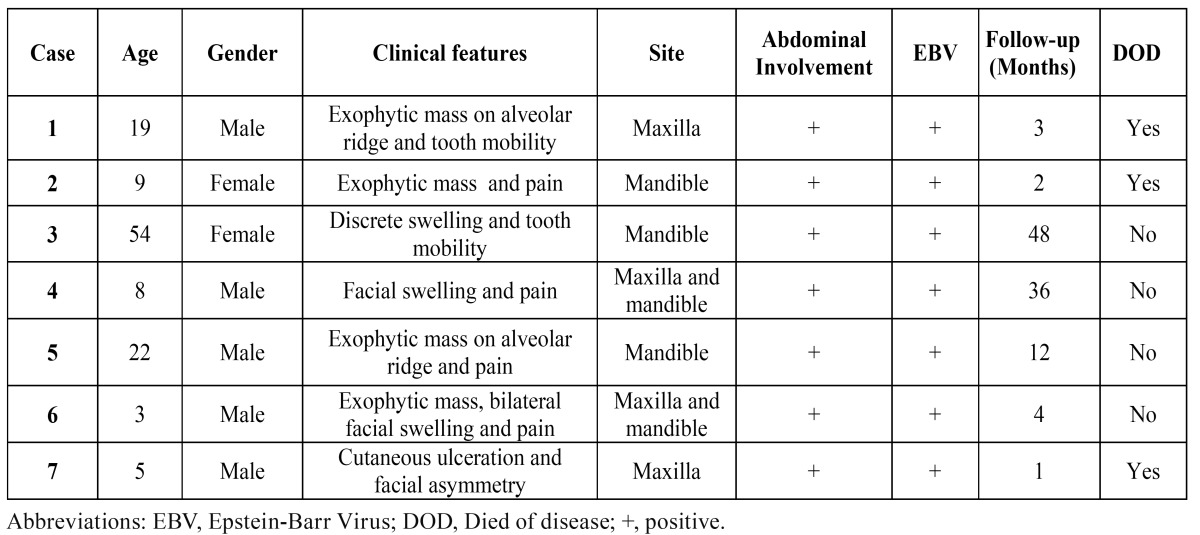
Clinical features of 7 patients with Burkitt’s lymphoma affecting the jaws.

**Figure 2:**
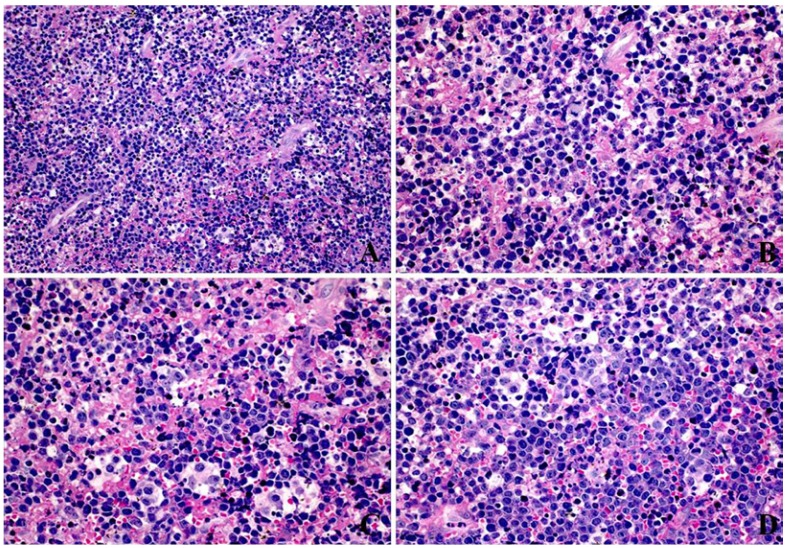
Histopathologic features of BL involving the jaws (Hematoxylin and eosin). A) Diffuse proliferation of medium-sized, non-cleaved neoplastic cells (200X). B) Mitotic figures can be identified throughout the specimen (400X). C and D) Variable amounts of macrophages giving the typical “starry sky” pattern seen in most cases. The neoplastic cells exhibited one to three evident nucleoli and scant basophilic cytoplasm (400X).

**Figure 3:**
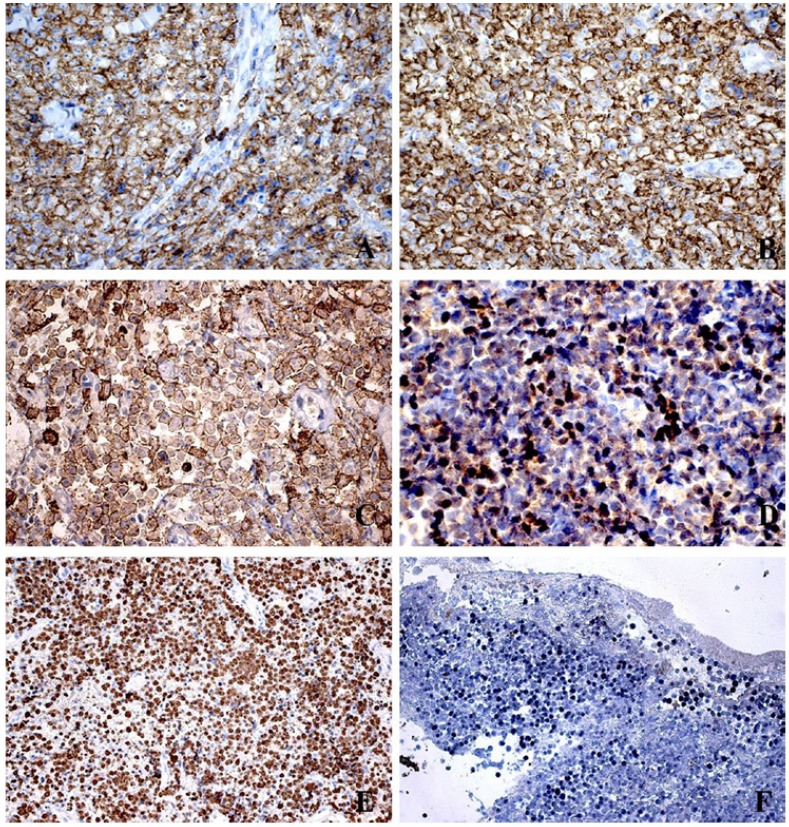
Immunohistochemical (Streptavidin-Biotin) and EBV detection (In situ Hybridization) in BL of the jaws. A) CD45 membrane staining (400X). B) Diffuse CD20 membrane staining (400X). C) CD10 positive membrane staining (400X). D) Bcl6 nuclear positivity (400X). E) High proliferative index measured by Ki67 nuclear staining (200X). F) EBV proved to be positive in all cases investigated (EBER; 200X).

Histologically all cases revealed the classic diffuse starry-sky like pattern of BL, with a diffuse proliferation of medium-sized neoplastic lymphoid cells, with scant basophilic cytoplasm and one to three evident nucleoli (Fig. 2). Immunohistochemically, all cases were positive for CD45, CD20, CD79a, CD10, Bcl6, with a very high proliferative index, close to 100% by Ki67 nuclear staining. All cases proved to be positive for EBV by ISH reactions (Fig. 3).

Six patients were treated with R-CHOP (Rituximab + Cyclophosphamide, Doxorubicin, Vincristine and Prednisolone) chemotherapy protocol, but one young adult patient refused to receive treatment and died of the disease three months later. Two patients died of the disease after one and two months after starting chemotherapy, whereas the other four patients are free of tumor, and under close follow-up.

## Discussion

BL is an aggressive B-cell lymphoma with a very high proliferative rate and rapid doubling time that reveals different clinical and biological characteristics mostly depending on the geographic region investigated (14,15). Brazil is a large country with distinct geographic areas. There are few series evaluating the clinicopathological features of BL in the Brazilian population, none emphasizing the description of BL with jaw involvement in the Amazon region, which presents environmental features similar to those found in equatorial Africa. In fact, the results of the present work suggest that BL with jaw involvement in the Brazilian Amazon region presents similar characteristics to endemic BL seen in Africa. Most of the cases affected infants, with simultaneous involvement of the jaws and abdomen, and all cases were positive for EBV.

BL in Brazil is considered of an intermediate type, clinically resembling sporadic BL, mostly presenting abdominal involvement and rarely affecting the jaws, with about 60% of the cases revealing positivity for EBV (16,17). In one of the largest studies of BL conducted in Brazil, 234 cases from all geographic regions of the country were analyzed, showing that in only 5 cases the jaws were affected (8). Similarly, in the 37 BL cases described by Klumb et al. (11) no case showed gnathic involvement, whereas the abdominal region was affected in 81% of the individuals. Abdominal involvement in most of the cases were also found by Hassan et al. (13) in 54 cases of BL from Rio de Janeiro and by Bacchi et al. (16) in 24 cases from São Paulo. Although the 7 cases described in the current study showed abdominal involvement, the jaws were also involved in all of them, similarly to endemic BL that are also EBV positive. Therefore, the characteristics of the cases here reported seem to be different from the general series described in Brazil that classifies BL as of an intermediate type, with abdominal involvement of adult patients.

It has been postulated that EBV is involved in the pathogenesis of BL, but the mechanisms involved are not yet fully understood (15,18). EBV might block the apoptosis of infected B cells with a MYC translocation through either the Epstein-Barr nuclear antigen 1 (EBNA1) protein, the Epstein-Barr virus BamHI fragment H rightward open reading frame 1 (BHRF1) protein, EBER transcripts, or epigenetic modiﬁcations and subsequent repression of pro-apoptotic proteins. The virus can also promote genomic instability, deregulate telomere functions and induce DNA damage to infected cells (5,6).

The relatively high EBV positivity in Brazilian cases, ranging from 50-84%, is consistent with an intermediate value between cases of equatorial Africa (100%) and North American BL (30%) (10,17-22). Gutierrez et al. (12) well illustrated this incidence by verifying that EBV was associated with BL from Southern Brazil in about 51% of the cases. However, different studies have shown that EBV positivity in Brazilian BL cases seems to be higher in the Northeast states than in the more developed Southeast areas. Hence, Araújo et al. (9) found 87% of EBV positivity in Northeastern region and Queiroga et al. (8) found significantly higher incidence of EBV positive cases in Northern region (76%) if compared to the Southern states (29%).

All cases in the present series revealed positivity for EBV, similar to endemic BL. In fact, the Brazilian Amazon region resembles equatorial Africa concerning its geographical characteristics, also presenting low socioeconomic level and high incidence of tropical diseases. In Equatorial Africa, BL has been associated with malaria, since Plasmodium falciparum is suggested to play a role in the development of this neoplasm (18,23). Hence, this association could also be eventually relevant in the Amazon area that is also known to be endemic for this infection. It would be important, therefore, to better determine the clinical and pathological characteristics, as well as the EBV status of a larger series of BL in Brazil, particularly with jaw involvement, adequately considering the broad differences present among all regions with especial attention for cases from the Amazon area.

Current therapeutic strategy for patients affected by BL includes the use of R-CHOP chemotherapy and the results obtained are in general highly satisfactorily particularly for children (6). In the present study, 2 patients out of 6 died of the disease after treatment and one patient did not receive treatment.

In conclusion, this series of 7 cases of BL involving the jaws of patients from the Amazon region of Brazil, most of them infants, indicates that they are similar to endemic BL of equatorial Africa, with all cases proving to be positive for EBV. Although it is well established that in Brazil most of the BL cases are considered of an intermediate type, with predilection for abdominal involvement and at about 60% of positivity for EBV, it would be interesting to analyze larger series of BL affecting patients from all different regions of Brazil, especially from the Amazon area to better clarify the findings of this study.
